# An electronic health record-based strategy to recruit for a Patient Advisory Council for Research: Implications for inclusion

**DOI:** 10.1017/cts.2019.433

**Published:** 2019-11-25

**Authors:** Nassira Bougrab, Dadong Li, Howard Trachtman, Scott Sherman, Rachel Thornton, Aisha T. Langford

**Affiliations:** 1 NYU Clinical and Translational Science Institute Recruitment and Retention Unit, NYU Langone Health, New York, NY, USA; 2 Clinic Informatics, Regeneron Pharmaceutics, Inc., Tarrytown, NY, USA

**Keywords:** Patient participation, electronic health records, International Classification of Diseases, medical informatics

## Abstract

In 2017, the NYU Clinical and Translational Science Institute’s Recruitment and Retention Unit created a Patient Advisory Council for Research (PACR) to provide feedback on clinical trials and health research studies. We collaborated with our clinical research informatics team to generate a random sample of patients, based on the International Classification of Diseases, Tenth Revision codes and demographic factors, for invitation via the patient portal. This approach yielded in a group that was diverse with regard to age, race/ethnicity, sex, and health conditions. This report highlights the benefits and limitations of using an electronic health record-based strategy to identify and recruit members for a PACR.

## Introduction

Historically, patient and family advisory councils were convened to enhance the user experience and patient and family support services in the context of clinical care [1–7]. In recent years, patient, family, and community advisory councils (PFACs) have been convened to help ensure that clinical trials and health research studies are designed with patients and communities in mind [8–12]. Often, these research-specific PFACs are created in the hope that they will improve the quality of research in general and recruitment and retention rates in particular [9,10,12].

Despite growing support for PFACs as a means of promoting patient engagement, the literature on the efficacy of PFACs across health care and research contexts remains limited [10,13]. In particular, there is a shortage of data regarding how health systems select patients to serve on PFACs, both for clinical care and research purposes, as well as which methodologies constitute best practice [4,10,13]. In 2018, Oldfield et al. published a systematic review of the PFACs in health care and research that included methods for recruiting PFAC members [10]. Of the 16 studies included in their review, most (*n* = 11) performed PFAC recruitment by nomination; the remainder (*n* = 5) did not describe their recruitment strategy. Common methods for identifying patients to serve on clinical care and research-related PFACs include nomination or referral, leveraging established community partnerships, word-of-mouth, and promotional flyers in clinics and waiting rooms [3,10,13]. The use of electronic health records for PFAC recruitment, however, remains underexplored.

Patient portals such as MyChart in Epic provide the opportunity to invite patients to serve on PFACs by sending direct messages. Patients generally hold positive attitudes about web messaging and use of electronic health records to facilitate patient–provider communication [14,15]. Randomly identifying patients via the electronic health record and directly inviting them to serve on PFACs through the patient portal may help to improve inclusion on PFACs from the standpoint of sociodemographic factors and health conditions. This approach may prevent the implicit or explicit biases associated with relying on health care professionals or community members as gatekeepers of invitations. In addition, personal invitations are more likely to be seen and read than indirect or impersonal recruitment methods such as flyers posted on a clinic bulletin board.

In 2015, the NYU Clinical and Translational Science Institute (CTSI) Recruitment and Retention Unit was established to assist study teams with the assessment of study recruitment feasibility and ways to maximize participant retention. In 2017, the Recruitment and Retention Unit created a Patient Advisory Council for Research (PACR) to provide feedback on clinical trials and health research studies as another resource for investigators.

### Objective

The objective of this brief report is to describe NYU CTSI’s process for identifying and recruiting patients to serve on a PACR through the electronic health record. Process measures and outcomes of our PACR will be reported in a forthcoming manuscript. Briefly, PACR members provide feedback on studies in the early development stages, including (1) perceived burden of being a patient in the study, (2) comprehensibility of the patient education materials to be used in the study, and (3) proper reimbursement based on study requirements. At each meeting, PACR members receive an $85 Visa gift card and $10 cash to help cover the cost of travel on public transportation. A boxed meal is also provided, as meetings occur during dinner hours.

## Materials and Methods

### Patient Identification

Our goal was to recruit approximately 16–20 PACR members, with the expectation that 10–15 members would attend each meeting. We used NYU Langone Health’s Epic database to generate a list of all patients with an active MyChart account between June 2017 and August 2018. Patients with a home address in New York, New Jersey, or Connecticut were included in the random sample. Each queried patient had a diagnosis of at least one of the following health conditions between January 1, 2010 and June 25, 2018: cancer, diabetes, cardiovascular disease, rheumatologic disease, neurologic disease, orthopedic disease, or renal disease.

We defined all health conditions based on the International Classification of Diseases, Tenth Revision (ICD-10) codes. We used stratified sampling techniques based on sex, race/ethnicity, and age group to select a final random sample of 1000 patients in which all 3 of these demographic features were in balanced proportions. To ensure age diversity, and because several studies at NYU Langone Health are focused on older adults, patients were divided into four age groups: 18–36 years, 37–55 years, 56–74 years, and 75–93 years. Race/ethnicity was categorized as Asian, Black, Hispanic, or White. After discussion with our IRB, it was determined that the creation of the PACR did not constitute a research study requiring IRB review, but rather a quality improvement. Consequently, it was not necessary to obtain informed consent from participants.

### MyChart Message Invitation

The following text is an abbreviated version of the invitation sent via the patient portal: “We are reaching out to patients of NYU Langone Medical Center who may have interest in serving on our new PACR. This council will provide feedback on ways to make it easier and more appealing for patients to participate in NYU clinical trials and health research studies. Our goal is to have a variety of ages, health conditions, sexes, and cultural groups represented on the council. If you are interested in serving on the PACR, you will be asked to: (1) Participate in six meetings per year to review research projects and recruitment strategies; (2) Give suggestions about ways to make research projects more patient-friendly; (3) Give suggestions on how to conduct research projects in doctors’ offices and in the community; and (4) Share your opinions about how to get the word out about the research projects.” Each patient received a single invitation.

Invitees were informed that they would receive a stipend for their time on the council, a meal at each meeting, and reimbursement for transportation. Patients who were interested in serving on the council were advised to reply to the message in MyChart. A phone number and email address for the CTSI Recruitment and Retention Unit program coordinator were also provided in the body of the message.

### Final Patient Selection for the PACR

The Recruitment and Retention Unit program coordinator followed up with all patients who expressed interest in serving on the PACR and scheduled a telephone interview. During the interviews, patients were asked about their experiences participating in group activities, either personally or professionally, their reasons for wanting to serve on the PACR, and health topics they thought might be interesting for NYU Langone Health researchers to study in the future. In addition to enabling the program coordinator to get to know the patients better, the interviews provided the program coordinator with the opportunity to further explain the purpose and structure of the PACR and to answer any remaining questions.

**Fig. 1.**
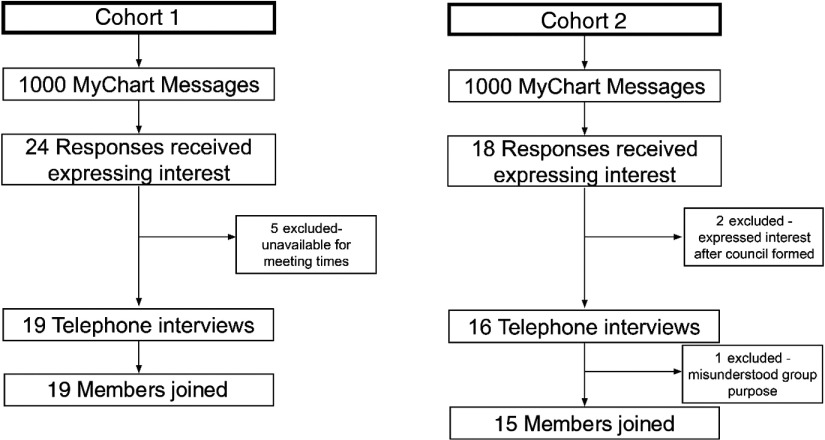
Recruitment process diagram.

The calls also enabled patients to elect to continue the process or to withdraw after learning more about the purpose and function of the group. For example, one patient thought that they were being asked to participate in a specific clinical trial related to a medical condition and declined to participate after the purpose of the council was further explained (see Fig. 1).

## Results

As shown in Table 1, patients were diverse with regard to sex, race/ethnicity, and age. The health conditions represented on the PACR included, but were not limited to HIV infection, multiple sclerosis, heart disease, hypertension, diabetes, osteoarthritis, rheumatoid arthritis, history of cancer, and fibromyalgia.

**Table 1.** Patient advisory council for research characteristics^+^

	Year 1 PACR members who joined in 2017 (*n* = 19)	Year 2 PACR members who joined in 2018 (*n* = 15)	PACR members as of January 1, 2019 (*n* = 25)^++^
Sex			
Female	7 (35)	9 (60)	14 (56)
Male	12 (63)	6 (40)	11 (44)
Race/Ethnicity			
Asian	2 (11)	1 (7)	2 (8)
Black	6 (32)	8 (53)	12 (48)
Hispanic	5 (26)	2 (13)	3 (12)
White	6 (32)	4 (27)	8 (32)
Age group, years			
18–36 years	2 (11)	4 (27)	6 (24)
37–55 years	6 (32)	2 (13)	3 (12)
56–74 years	7 (37)	7 (47)	12 (48)
>75	4 (21)	2 (13)	4 (16)

PACR, Patient Advisory Council for Research.

+ These numbers include those who initially responded and agreed to be on the council. Actual attendance varied by meeting date.

++ Current PACR members as of January 1, 2019 include members from Year 1 who agreed to serve another year and new members invited in Year 2.

## Discussion

The purpose of this brief report was to describe a method for using the electronic health record to identify and recruit patients to serve the NYU CTSI’s PACR. Patient portals have been used to invite patients to participate in clinical trials and health research studies [16], as well as for clinical care purposes [17,18]. However, we are unaware of other institutions using this approach for PFAC recruitment. We found an electronic health record-based approach to be feasible and broadly inclusive of various health conditions and sociodemographic groups. Accordingly, patient portals may hold promise for reaching a wider audience of PACR participants than traditional methods such as nomination or flyers, but this hypothesis needs to be tested in future work.

Although the response rate for joining our PACR was relatively low (<5%), this number should be viewed in context. First, given how invitations were sent (a direct message in MyChart), we do not know how many patients actually opened their invitations, or whether the email addresses on record to alert patients of a new message in MyChart were still being utilized at the time of invitation. Second, agreeing to serve on the PACR required the following: (1) the ability to travel to mid-town Manhattan on a Wednesday evening six times a year, (2) the willingness to spend two hours at each meeting, plus travel time to and from the meeting, and (3) the willingness to share thoughts openly in a group setting. We do not know how many invitees would have been interested in participating on the PACR in general but could not make the prespecified meeting times, as opposed to being altogether uninterested in participating. Nevertheless, we reached our desired number of PACR members using this approach.

### Strengths, Limitations, and Future Directions

The strengths of our method included the ability to randomly invite a large pool of patients based on predetermined criteria across all health system clinics, thereby increasing the likelihood that we would recruit PACR members with a variety of health conditions and from different sociodemographic groups. Despite these advantages, the approach has some limitations. Not all patients in our health system have an active patient portal account (i.e., MyChart). Hence, we may have missed patients who would have been interested, but would need to be contacted by phone, mail, email, or text message. The overall diversity of patient portal users can be limited with regard to race/ethnicity, sex, literacy, language spoken, and socioeconomic status [14,15]. Hence, the construction of the PACR may have been different if we had used recruitment methods beyond the patient portal. We selected ICD-10 codes that reflected common conditions in the general population, but patients with other health conditions or rare diseases may have been interested in serving on the PACR. Finally, although our invitation process was random, the final selection of PACR members was influenced by patients’ ability to attend on the day and time chosen for council meetings.

Future research should compare different methods for inviting participants to join PFACs (e.g., patient portal, letter, nomination, and flyers). To date, no study has reported a head-to-head comparison of methods for inviting patients to serve on PFACs. Such a study could help to determine whether the invitation method influences esponse and long-term participation rates.

## Conclusion

By randomly identifying patients from our electronic health record system and then directly inviting them to join our PACR via the patient portal, we were able to create a group that was broadly inclusive of various health conditions and sociodemographic factors. Further research should seek to validate the efficacy of this method for creating PFACs. In particular, more research is needed to better understand who is being contacted through an electronic health record-based strategy, and which patient subgroups may be inadvertently yet systematically excluded by such approaches.
